# Ophthalmologic Manifestations and Retinal Findings in Children with Down Syndrome

**DOI:** 10.1155/2020/9726261

**Published:** 2020-02-07

**Authors:** Adem Ugurlu, Emre Altinkurt

**Affiliations:** ^1^Erzincan Binali Yildirim University, Faculty of Medicine, Department of Ophthalmology, Erzincan 24100, Turkey; ^2^Istanbul University, Faculty of Medicine, Department of Ophthalmology, Istanbul 34104, Turkey

## Abstract

**Purpose:**

To evaluate ocular findings in children with Down syndrome and to compare with the healthy children group.

**Methods:**

The study patients were divided into two groups as the diagnosed Down syndrome group and the control group. The study was designed as a prospective and single-center study in Istanbul University Faculty of Medicine Department of Ophthalmology. The study included 93 patients in the age range from 7 to 18 years, who applied to the ophthalmology department of our clinic in the period from July 2017 to June 2018. The study included the patients allocated into the control group and the Down syndrome patients allocated into the patient group, containing 49 and 44 participants, respectively. All patients underwent complete ophthalmologic examination with biomicroscopy. Autorefractometer measurements were performed in all patients, and the best corrected visual acuity (BCVA) was determined with the use of the Snellen chart. All patients underwent spectral domain optical coherence tomography (SD-OCT) measurements for central foveal retinal (CRT), subfoveal choroidal (CCT), and peripapillary retinal nerve fiber layer (pRNFL) thicknesses.

**Results:**

The average CRT was 241.2 ± 25.7 microns in Down syndrome group and 219.4 ± 21.1 microns in the control group. There was a statistically significant difference between the groups in regards to CRT (*p* < 0.001). The average pRNFL values were 123.1 ± 15.4 microns in the Down syndrome group and 102.2 ± 8.7 microns in the control group (*p* < 0.001). The average pRNFL values were 123.1 ± 15.4 microns in the Down syndrome group and 102.2 ± 8.7 microns in the control group (

**Conclusions:**

In the subjects with Down syndrome, the incidence of lens opacities, strabismus, and amblyopia was higher than the control group. CRT and pRNFL were thicker in the Down syndrome group than in control group. This may represent retinal developmental changes in the patients with Down syndrome.

## 1. Introduction

Down syndrome, also known as trisomy 21, is a genetic disorder caused by the presence of an extra chromosome 21 as a whole or by the presence of its copied parts in addition to the pair present in the normal human genome [1, 2]. The syndrome is typically associated with delays in the physical growth, characteristic facial features, and mild-to-moderate intellectual disability [3]. Down syndrome is also frequently associated with a wide range of ocular complications including refractive errors, eyelid abnormalities, strabismus, nystagmus, abnormalities in the tear duct and the iris, the presence of keratoconus, and congenital or developmental cataracts [4–11].

An early ophthalmic examination is essential for the early treatment of the diseases of the eye [7–9]. It is not easy to diagnose eye pathologies in children with Down syndrome because of the difficulties which may arise during an eye examination.

With an early and accurate diagnosis, certain eye pathologies can be treated and visual rehabilitation can be provided [12, 13]. Improvements in the vision allow individuals with Down syndrome to integrate into the social life easily [12–15]. This is why the visual treatment of people with Down syndrome is important for both the individual and the parents, as well as it is for the community [15].

The aim of our study was to examine the ocular findings in children with Down syndrome in the age range from 7 to 18 years and to evaluate these findings in comparison to the normal population in the same age group.

## 2. Subjects and Method

### 2.1. Ethical Approval

The study was conducted in compliance with the principles of the Declaration of Helsinki. Approval was obtained from the Institutional Review Board of Istanbul University Faculty of Medicine. Informed consent was obtained from the parents of all patients.

### 2.2. Settings and Participants

The study was designed as a prospective and single-center study. The study included 93 patients in the age range from 7 to 18 years, who applied to the ophthalmology department of our clinic in the period from July 2017 to June 2018. None of the study patients had a previous history of ocular surgery. The study included the patients allocated into the control group and the Down syndrome patients allocated into the patient group, containing 49 and 44 participants, respectively. All of the patients with Down syndrome had normal gestational age such as in the control group. The control patients were recruited from children who were evaluated in our clinic, and they had not any ocular disorders such as corneal disease, cataract, glaucoma, retinal pathology, uveitis and ambliyopia. Control group patients had only refractive and refractive disorders related problems such as strabismus and these are recorded. The participants were excluded from the study when they failed to comply with the examination schedule. Patients with optic nerve head drusen in the Down syndrome group were excluded when analyzing pRNFL difference between two groups.

### 2.3. Ocular Examinations and OCT Measurements

Auto refractometer (KR-8900, Topcon, Tokyo, Japan) measurements were performed in all patients and the best corrected visual acuity (BCVA) was determined with the use of the Snellen chart, the results of which were then converted to LogMAR. The regular calibration of autorefractometer was performed. Hiperopia over 0.50 diopter and miyopia under 0.50 diopter were accepted as hiperopia and miyopia in both groups in the study. All patients were examined for strabismus and eye movements. A detailed examination of the anterior segment of the eye with biomicroscopy and intraocular pressure measurements with applanation tonometers were performed in all patients, and findings were recorded. After dilating the pupil with the use of a combination of tropicamide %1 (Tropamid®, Bilimilaç®, Gebze, Turkey) and phenylephrine hydrochloride %2.5 (Mydfrin®, Alcon®, Fort Worth, TX) in all patients; refractive status was examined under cycloplegia and the fundoscopic examination was performed with 90 D fundus lens under biomicroscopy. Cycloplegia was considered complete with the absence of light reflex and pupil diameter more than 6 mm. SD-OCT (spectral domain optical coherence tomography) and RNFL (retinal nerve fiber layer) measurements were performed with a Heidelberg spectralis domain OCT device (Heidelberg Engineering®, Germany). All patients underwent SD-OCT and RNFL scans for the measurements of the central foveal retinal (CRT), subfoveal choroidal (CCT), peripapillary retinal nerve fiber layer (pRNFL), and inner retinal layer (IRL) thicknesses. To evaluate the choroidalscleral interface, the Spectralis EDI (enhanced depth imaging) setting was used in the study. The subfoveal choroidal measurements were taken from three points in the OCT scans of the patients. The first measurement was performed in the central subfoveolar area. The second measurement was performed in a point 500 microns away from the subfoveolar area towards the nasal side. Finally, the third measurement was made 500 microns away from the subfoveolar area towards the temporal side. The mean of the values obtained from these points was recorded for all patients. To minimize diurnal variation of the subfoveal choroidal thickness, the SD-OCT examinations were conducted by one experienced examiner between 10:00 a.m. and 3:00 p.m. each day. All OCT measurements were evaluated by an experienced ophthalmologist and CRT, CCT, and IRL measurements were calculated manually and recorded. CRT measurement was calculated by measuring the length of the line drawn perpendicular to the RPE-Bruch membrane complex from the midpoint of the foveola and IRL measurement by measuring the length of the line drawn perpendicular to the outer plexiform layer from the midpoint of the foveola. The definition of CCT was the vertical distance between Bruch's membrane that is under the projection of foveola and the choroidalscleral interface.

### 2.4. Statistical Analysis

The Kolmogorov–Smirnov test was applied to observe the distribution of the parameters in the study groups. Continuous variables were expressed as mean ± standard deviation (SD), and categorical variables as frequencies and percentages. The chi-square test was used for comparing the nominal data. The independent *t*-test was used as a parametric test for comparing normally distributed data and the Mann–Whitney-U analysis was used as a nonparametric test. Pearson correlation analysis was used for evaluating the data correlations between the two groups. Exact *p* values of <0.05 were considered statistically significant. IBM SPSS (Statistical Package for the Social Sciences). Statistics 22 program was used in the statistical analyses.

## 3. Results

### 3.1. General Characteristics of the Participants and Difference between Gender and Age

The study patients were divided into two groups as the diagnosed Down syndrome group and the control group. The demographic features and the ophthalmic examination outcomes of the study patients can be seen in Table 1. The study included a total of 44 right eyes of 44 patients diagnosed with Down syndrome in the age range from 7 to 18 years in the Down syndrome group and 49 right eyes of 49 patients in the same age range in the control group. In Down syndrome group, 21 patients were males and 23 patients were females. In the control group, 24 patients were males and 25 patients were females. The gender difference was not different between the groups (*p*=0.798). The mean age was 13.10 ± 3.20 years in the Down syndrome group, and it was 12.18 ± 3.32 years in control group. There was not a statistically significant difference in age between the groups (*p*=0.090).

### 3.2. Visual Outcomes and Refractive Status

The mean BCVA value was 0.19 ± 0.03 (±SE) LogMAR in the Down syndrome group and 0.005 ± 0.004 (±SE) LogMAR in the control group. There was a significant difference between the two groups in the BCVA values (*p* < 0.001). The mean intraocular pressure was 13.4 ± 1.9 mmHg in the Down syndrome group, and it was 12.5 ± 3.1 mmHg in the control group. There was not a statistically significant difference in the mean intraocular pressure between the groups (*p*=0.124). In the Down syndrome group; 31 of 44 (70.5%) subjects were hyperopic, and 13 of 44 (29.5%) subjects were myopic. In the control group, 34 of 49 (69.4%) subjects were hyperopic, and 15 of 49 (30.6%) eyes were myopic. There were no statistically significant differences in the spherical refractive errors between the two groups (*p*=0.934). Astigmatism over 1 diopter was found in 31 out of 44 subjects in the Down syndrome group and in 12 out of 49 subjects in the control group. The difference in astigmatism degrees was significantly different between the study groups (*p* < 0.001).

### 3.3. Orthoptic and Biomicroscopic Examinations

**Figure 1:**
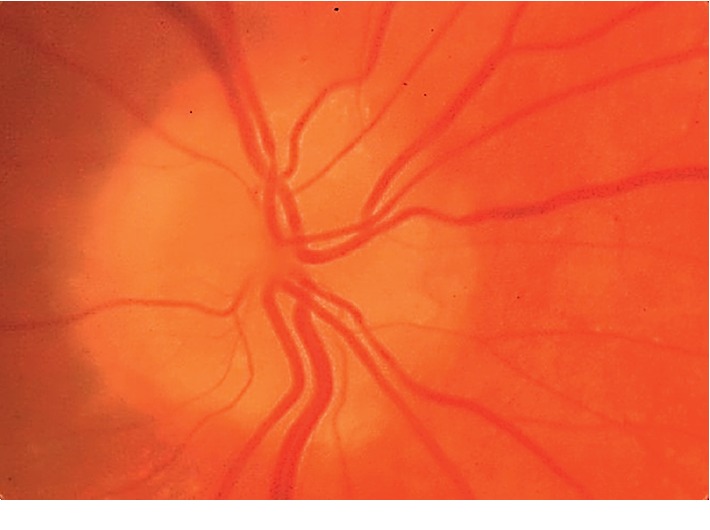
Optic nerve head drusen in a patient with Down syndrome in the study.

**Figure 2:**
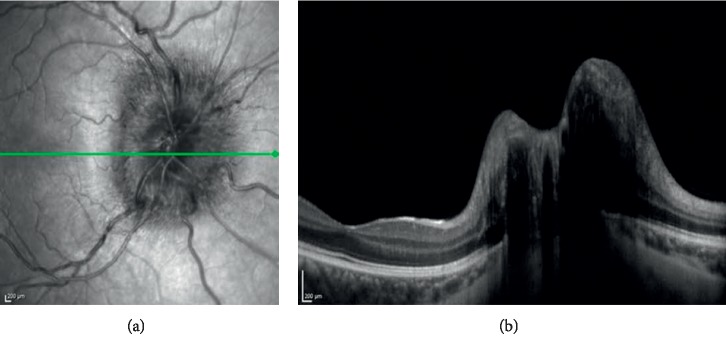
ONHD in OCT in a patient with Down syndrome.

Table 1 shows the outcomes of the orthoptic and biomicroscopic examinations. In the Down syndrome group, 12 eyes (27.3%) had blepharitis while 4 eyes (8.2%) had blepharitis in the control group. The difference was significantly different between the groups (*p* < 0.001). When the patients were evaluated for the lens opacities; 14 eyes in the Down syndrome group and none of the eyes in control group had lens opacities, demonstrating a significant difference between the two groups (*p* < 0.001). Strabismus was detected in 10 patients (8 with esotropia, 2 with exotropia) (22.7%) in the Down syndrome group and in 4 patients (2 with esotropia, 2 with exotropia) (8.2%) in the control group. The difference was statistically significant (*p* < 0.001). The types of strabismus were accomodative (refractive) esotropia and intermittent exotropia in the control group. In the Down syndrome group, only one patient had nystagmus, and in the control group there were no patients with nystagmus. The frequencies of nystagmus were not statistically significantly different between the groups (*p*=0.343). There were 16 amblyopic eyes in the Down syndrome group. In the control group, there was not any amblyopic eye. In the Down syndrome group, the amblyopia rate (36.4%) was significantly higher compared to that in the control group (*p* < 0.001). In the Down syndrome group, 12 subjects (27.3%) had Brushfield spots in the iris, leading to a significant difference between the two groups (*p* < 0.001). Optic nerve head drusen (ONHD) was seen in 3 eyes (6.8%) in the Down syndrome group. (Figures 1 and 2). These lesions were not detected in any of the eyes in the control group. There was a statistically significant difference in the frequency of ONHD between the study groups (*p* < 0.001).

### 3.4. OCT Measurement Outcomes

The outcomes of the OCT scan imaging are presented in Table 2. The mean central foveal retinal thicknesses (CRT) was 241.2 ± 25.7 microns in the Down syndrome group, and it was 219.4 ± 21.1 microns in the control group. There was a statistically significant difference between the groups in regards to CRT (*p* < 0.001). There was a moderate positive correlation between BCVA and CRT as a result of Pearson correlation analysis (*p* < 0.001, *r*: 0.548). Choroidal measurements were performed with the Spectralis EDI-OCT setting. In the Down syndrome group, the mean subfoveal choroidal thickness was 336.4 ± 48.7 microns. In the control group, the mean subfoveal choroidal thickness was 334.1 ± 56.2 microns. In regards to the mean subfoveal choroidal thickness, there was not a statistically significant difference between the groups (*p*=0.728). The mean pRNFL values were 123.1 ± 15.4 microns in the Down syndrome group and 102.2 ± 8.7 microns in the control group (*p*=0.001). Furthermore, all quadrants of pRNFL were thicker in the Down syndrome group than in the control group. There was a significant difference between the two groups in terms of the measured pRNFL values (*p* < 0.001). The mean IRL thicknesses were 49.3 ± 6.4 microns in the Down syndrome group, and it was 25.1 ± 4.9 microns in the control group. There was a statistically significant difference between the groups in regards to IRL (*p* < 0.001).

## 4. Discussion

In our study, the frequencies of lens opacities, strabismus, blepharitis, and amblyopia are higher as expected in the patient group with Down syndrome than in the control group, which is a finding in line with the information in the literature [16, 17]. Due to the high incidence of strabismus, amblyopia, and cataracts in the patients with Down syndrome, BCVA values were significantly lower in the Down syndrome group than those found in the control group [18].

The CRT and pRNFL thickness were higher in the Down syndrome group than the values in the control group as determined by means of the measurements in the SD-OCT images.

Laguna et al. showed that the RNFL was significantly thicker in patients with Down syndrome than in the control group [19]. In the same study, it was reported that the sensorial retinal inner layers were thicker in trisomic mouse models than the thickness in the control group [19]. It has been reported that these findings might indicate differences in the retinal development, being associated with abnormal retinal development and with the presence of caspase-9-mediated apoptosis disorder [19].

O'Brien et al. determined that the central foveal retina was significantly thicker in the patient group with Down syndrome than the thickness found in the control group in their study, which is a consistent finding with the data obtained in our study [20]. The authors suggested that this condition might be associated with macular developmental disorders in patients with Down syndrome [20].

**Figure 3:**
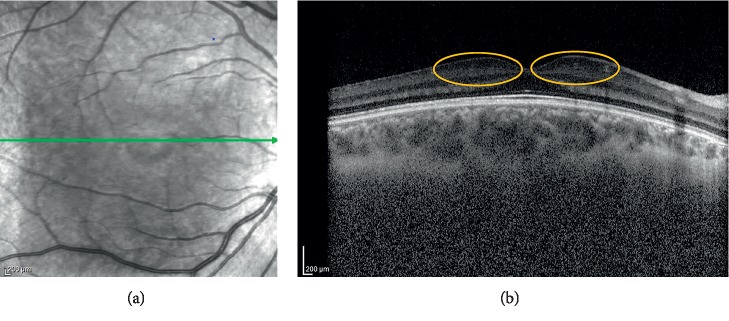
OCT shows thickening in inner segments of the central foveal retina (orange circles) in the patient with Down syndrome.

**Figure 4:**
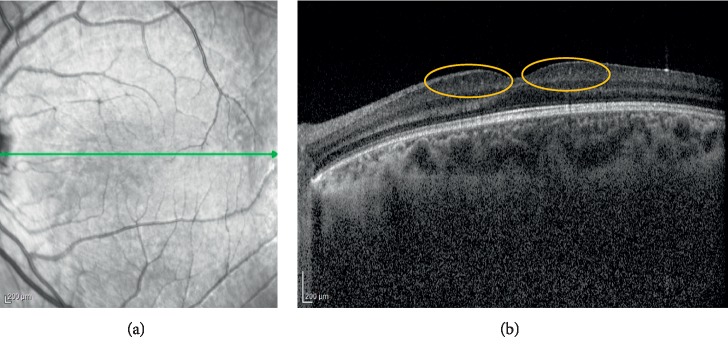
Thickening of inner retinal layers (orange circles) in OCT in another patient with Down syndrome.

Mangalesh et al. showed that babies with Down syndrome have abnormal foveal morphology and persistence of inner retinal layers [21]. In our study, the IRL measurements were thicker in the patients with Down syndrome, and this may be due to the persistence of inner retinal layers in the patients with Down syndrome (Figures 3 and 4). More studies with more patients with Down syndrome are needed to have an idea about the structural retinal changes in Down syndrome.

Schneier et al. showed that the incidence of ONHD was significantly higher in patients with Down syndrome [22]. In our study, the frequency of ONHD was higher in the children with Down syndrome than the value found in the normal population (Figures 1 and 2). This condition may occur due to a separate developmental problem in the RNFL in patients with Down syndrome.

Tomita evaluated visual characteristics of children with Down syndrome and showed that visual acuity reduction can be found despite a normal ocular exam. Refractive errors and visual developmental delays may cause vision loss in children with Down syndrome [23].

There was not a difference in the central subfoveal choroidal thickness between the two groups in our study. This suggests the presence of a difference in the neurosensorial development rather than that in the vascular development in individuals with Down syndrome.

Liu et al. investigated the effect of corneal astigmatism on retinal nerve fiber layer thickness in their study [24]. They compared the normal corneal astigmatism group with the higher corneal astigmatism group, and there were no significant differences between the two groups in global average RNFL thickness, as well as superior, nasal, and inferior quadrant RNFL thickness [24].

One of the limitations of the study is that the mental and cognitive status of the patients with Down sydrome was not questioned. The relationship between the mental and cognitive status and the retinal findings may be investigated in further research studies. Since there was nystagmus in one patient in the Down syndrome group, more patients were needed to tell whether there is a difference in the presence of nystagmus between the two groups. Another limitation of our study is that there was a difference in terms of astigmatism between two groups. Although there are publications in the literature that astigmatism may not affect RNFL measurements [24, 25], the significant difference between the two groups in terms of astigmatism is the limitation of our study.

## 5. Conclusion

This present study shows that the incidence of several specific eye diseases is increased in individuals with Down syndrome. Awareness of pediatricians and ophthalmologists about the Down syndrome may help patients with Down Syndrome to have a better vision and quality of life.

## Figures and Tables

**Table 1:** Demographic data and examination outcomes of patients.

Variables	Down syndrome	Control	Total	*p* value
Number (subjects)	44 (47.3%)	49 (52.7%)	93 (100%)	—
Age (years)	13.10 ± 3.20	12.18 ± 3.32	12.53 ± 3.29	0.090
Gender (men/women)	21 (47.7%)/23 (52.3%)	24 (49%)/25 (51%)	45 (48.4%)/48 (51.6%)	0.798
IOP	13.4 ± 1.9	12.5 ± 3.1	13.3 ± 1.7	0.124
BCVA	0.19 ± 0.03	0.005 ± 0.004	0.07 ± 0.03	**<0.001**
Hyperopia	31/44 (70.5%)	34/49 (69.4%)	65/93 (69.9%)	0.891
Myopia	13/44 (29.5%)	15/49 (30.6%)	28/93 (30.1%)	0.934
Astigmatism	31/44 (70.5%)	12/49 (24.5%)	43/93 (46.2%)	**<0.001**
Blepharitis	12/44 (27.3%)	4/49 (8.2%)	16/93 (17.2%)	**<0.001**
Lens opacity	14/44 (31.8%)	0/49 (0%)	14/93 (15.1%)	**<0.001**
Strabismus	10/44 (22.7%) 8 esotropia 2 exotropia	4/49 (8.2%) 2 esotropia 2 exotropia	14/93 (15.1%) 10 esotropia 4 exotropia	**<0.001**
Nystagmus	1/44 (2.3%)	0/49 (0%)	1/93 (1.1%)	0.343
Brushfield spot	12/44 (27.3%)	0/49 (0%)	12/93 (12.9%)	**<0.001**
Ambliyopia	16/44 (36.4%)	0/49 (0%)	16/93 (17.2%)	**<0.001**
ONHD	3/44 (6, 8%)	0/49 (0%)	3/93 (3.2%)	**<0.001**

IOP: intraocular pressure. BCVA: best corrected visual acuity. ONHD: optic nerve head drusen.

**Table 2:** OCT scan measurements of patients.

OCT outcomes	Down syndrome	Control	*p* value
CRT	241.2 ± 25.7	219.4 ± 21.1	**<0.001**
IRL	49.3 ± 6.4	25.1 ± 4.9	**<0.001**
CCT AVE	336.4 ± 48.7	334.1 ± 56.2	0.728
CCT SUBFOV	334.5 ± 49.7	330.1 ± 58.8	0.853
CCT 500M NAS	328 ± 53.3	334.7 ± 57.2	0.591
CCT 500M TEM	346.6 ± 44.8	337.5 ± 53.8	0.185
pRNFL AVE	123.1 ± 15.4	102.2 ± 8.7	**<0.001**
pRNFL TEM	100.3 ± 4.4	75.9 ± 15.3	**<0.001**
pRNFL NAS	88.1 ± 15.6	74.1 ± 14.6	**<0.001**
pRNFL TEMSUP	174.9 ± 33.6	141.1 ± 16.7	**<0.001**
pRNFL NASSUP	129.2 ± 17.2	113.4 ± 23.1	**<0.001**
pRNFL TEMINF	175 ± 29.6	144.6 ± 16.2	**<0.001**
pRNFL NASINF	133.1 ± 10.5	115.5 ± 24.5	**<0.001**

CRT: central foveal retinal thickness. IRL: inner retinal layer CCT: central choroidal thickness. AVE: average. SUBFOV: subfoveola. 500M: 500 micron. NAS: nasal. TEM: temporal. pRNFL: peripapillary retinal nerve fiber layer. TEMSUP: temporal superior. NASSUP: nasal superior. TEMINF: temporal inferior. NASINF: nasal inferior.

## Data Availability

The data used to support the findings of this study are available from the corresponding author upon request.
